# The influence of collecting patient-reported outcome measures on visit satisfaction in rheumatology clinics

**DOI:** 10.1093/rap/rkaa046

**Published:** 2020-09-01

**Authors:** Ryan Honomichl, Irene Katzan, Nicolas Thompson, Abby Abelson, Chad Deal, Susannah Rose, Brittany Lapin

**Affiliations:** Department of Quantitative Health Sciences; Neurological Institute Center for Outcomes Research & Evaluation; Department of Quantitative Health Sciences; Center for Osteoporosis and Metabolic Bone Disease Orthopedic; Department of Rheumatic and Immunologic Diseases; Department of Rheumatic and Immunologic Diseases; Office of Patient Experience, Cleveland Clinic, Cleveland, OH, USA; Department of Quantitative Health Sciences

**Keywords:** patient-reported outcomes, patient satisfaction, rheumatology

## Abstract

**Objectives:**

Patient-reported outcome measures (PROMs) can direct patient-centred care and increase patient satisfaction with the visit. The objective of this study was to assess the relationship between the collection of PROMs and visit satisfaction, as measured by the Clinician and Group Practice Consumer Assessment of Healthcare Providers and Systems (CG-CAHPS) survey.

**Methods:**

An electronic platform for collection of patient-reported information was implemented in rheumatology clinics between August and September 2016. Adult patients were included in the study if they completed CG-CAHPS after an ambulatory visit. The pre-implementation cohort consisted of patients seen between January and June 2016; the post-implementation cohort consisted of patients seen between January and June 2017. The CG-CAHPS scores were compared between cohorts. Mixed effect models were constructed to identify predictors of visit satisfaction.

**Results:**

Characteristics were similar between the 2117 pre- and 2380 post-implementation patients. Visit satisfaction was high in both cohorts but did not differ [odds ratio = 0.97 (95% CI: 0.79, 1.19)]. Predictors of improved satisfaction included being an established patient, being male, older age and reporting higher quality of life. However, sensitivity analyses in the post-implementation cohort suggested that implementing PROMs might convey benefits for new patients, in particular.

**Conclusion:**

Collection of PROMs had no effect on visit satisfaction in rheumatology clinics, although there might be benefits for new patients. These largely null findings might be attributable to high satisfaction levels in our cohorts or to lack of provider review of PROM data with patients. Further research is indicated to determine the impact of provider communication of PROM results to patients on different domains of visit satisfaction.

**Key messages**

- Patient-reported outcome measures may influence office visit satisfaction for patients seen in rheumatology clinics.
- Predictors included being an established patient, being male, older age and better quality of life.
- Patient-reported outcome measures may lead to improved provider ratings by enhancing communication, particularly in new patients.

## Introduction

Patient-reported outcome measures (PROMs) are progressively becoming a standard tool in research and clinical practice, taking an increasingly important role in clinical management and evaluation of patient outcomes [1–3]. Among the potential benefits of PROMs are improved symptom monitoring and management [4, 5], detection of undiagnosed psychological or functional issues [6], and greater patient engagement and satisfaction [7]. To maximize the effectiveness of PROMs, feedback from the physician is an important component [8]; however, merely completing measures might convey some benefits [9].

Previous research suggests that clinical practice in rheumatology might be well suited to benefit from the use of PROMs. PROMs might be especially applicable for patients with conditions that are more subjective in nature, such as rheumatological conditions. Clinical outcomes, such as laboratory values, are not always suitable, whereas patients’ perceptions of symptoms and the effect of the illness on their lives should be assessed [10] because rheumatology patients have been shown to differ from their physician in assessment of disease severity [11]. Furthermore, completing PROMs and discussing them with their physician might contribute to patients’ thinking more broadly about their condition and how it affects their life [12]. When incorporated into an office visit, PROMs have the potential to aid clinicians in better understanding health from the patient’s viewpoint, leading to enhanced communication and shared decision-making, and empowering the patient to self-manage their symptoms [13, 14]. Given this, incorporation of the patient’s perspective during treatment is paramount to increase patient engagement and enhance patient–provider communication [15].

A recent analysis by our group demonstrated that neurological patients completing PROMs as part of routine clinical care found the measures useful and reported improved communication with their provider [16]. In another study by our group, we found that patients reporting a more positive PROMs experience also reported higher overall visit satisfaction, as measured by the Clinician and Group Consumer Assessment of Healthcare Providers and Systems (CG-CAHPS) survey [17, 18]. The Center for Medicare & Medicaid Services ties Medicare reimbursements with patient satisfaction, as measured by the CG-CAHPS, which has implications for national rankings and quality metrics. Given the potential benefits of using PROMs in the rheumatology population, exploring the effect of completing PROMs on overall patient satisfaction with their office visit is of interest. Although our prior study and other correlational studies have examined the relationship between experience with PROMs and visit satisfaction, few studies have compared satisfaction between cohorts who did *vs* did not complete PROMs. Rheumatology centres at Cleveland Clinic were in a unique position to facilitate this comparison, because an electronic platform for the systematic collection of PROMs was implemented starting in August 2016. The objective of our study was to assess the influence of the collection of PROMs on overall patient satisfaction ratings through a comparison of CG-CAHPS scores pre- *vs* post-implementation of PROMs collection.

## Methods

This was a retrospective pre–post cohort study conducted within 13 ambulatory clinics in the Rheumatology Department at Cleveland Clinic. Inclusion criteria for participation was all adult (≥18-year-old) patients who completed CG-CAHPS surveys after their office visit. Electronic collection of PROMs was implemented within the Rheumatology Department between August and September 2016. Data were compared between a pre-implementation cohort of patients seen between January and June 2016 and a post-implementation cohort seen between January and June 2017. These windows were selected to account for effects of workflow transition owing to implementation of PROMs and to eliminate any effects of seasonality. The study was approved by the Cleveland Clinic Institutional Review Board. Given that the study consisted of analyses of pre-existing data, the requirement for patient informed consent was waived.

### Patient-reported outcomes

During routine care after implementation of PROMs, rheumatology patients completed the Patient Health Questionnaire 9 (PHQ-9) [19], the Patient-Reported Outcomes Measurement Information System (PROMIS) global health, fatigue, pain interference and physical function scales [20] and the condition-specific RAPID3 [21]. All PROMs were administered through Cleveland Clinic’s Knowledge Program (KP) [22]. The KP is a flexible health-care information technology platform that is currently used to collect patient-entered data in ∼115 000 patients each month within the Cleveland Clinic Health System. All patients completed measures on an electronic tablet immediately before their visit or at home before the visit via a patient portal (MyChart; Epic Systems, Epic, Verona, WI). PROMs data are integrated within the electronic health record and are available to the provider at the point of care. Providers acknowledge review of the PROM data by clicking a review/approve button. Although the majority of providers indicate review of the PROM scores, the extent to which they digest the data or discuss them with their patients is unknown. As such, our study does not have documentation of whether such a review and/or discussion took place during the visit.

### Patient satisfaction

The CG-CAHPS survey [23] is a standardized measure used to assess patients’ outpatient medical appointment experience. It is sent to a randomly selected sample of patients after medical practice visits through paper and email distribution methodologies. For the present study, primary outcomes included the overall provider rating on a 10-point scale (0 = worst doctor possible; 10 = best doctor possible), the patient’s report of willingness to recommend the caregiver to family and friends (response options: yes, definitely; yes, somewhat; no), and a composite measure of overall satisfaction with care. This composite measure consisted of provider rating of 9 or 10 and a highest possible response to each of the following items: provider listened; showed respect; and spent enough time [18]. Secondary outcomes included six items related to the Communication with Caregiver domain.

### Demographics and clinical characteristics

Patient demographics were collected, including age, race, marital status, household income estimated from 2010 census data by zip code, and patient history of co-morbidities. Additionally, a Charlson co-morbidity index (CCI) was also available. The CCI reflects 19 conditions related to the potential for mortality and morbidity [24]. Finally, a variable was also included to indicate whether the patient was new to the Rheumatology Department at the time of the study visit or whether they were an established patient.

### Statistical analysis

Descriptive statistics were used to summarize and compare demographics and clinical characteristics between the pre-implementation and post-implementation cohorts. A frequency count with percentage was used to present categorical data, and mean with s.d. or median with interquartile range was used to present continuous data. Chi-square tests were used to compare categorical variables, and Student’s unpaired *t*-tests (parametric) or Mann–Whitney *U* tests (non-parametric) were used to compare continuous variables across cohorts, as appropriate. Unadjusted odds ratios (ORs) were calculated from univariate mixed effects logistic regression models, to account for possible repeated measures from patients seen in both cohorts, for top box responses to each of the six communication domain items, the probability of a score of 9 or 10 on the provider rating scale, a recommendation of the provider’s office to family/friends, and the composite measure of satisfaction with care. The models examined the effect of cohort (post- *vs* pre-implementation of PROMs).

Subsequently, multivariable models were adjusted for demographic and clinical covariates identified *a priori*: whether the patient was new to Rheumatology, age, sex, marital status, race, income, education level, Charlson score, and a single item on quality of life (QOL) from the CG-CAHPS. For QOL, a low score indicator was calculated for patients who did not respond with excellent or very good. Health-care provider was also included as a random effect. All possible two-way interaction terms with cohort were introduced to these models to determine whether there was a differential effect of completing PROMs on outcomes. Statistical significance was established throughout at *P* < 0.05. Given that the results of this data analysis are exploratory and hypothesis generating, no formal adjustment for multiple comparisons was made. Missing data were minimal; therefore, no imputation was conducted. All analyses were completed in R v.3.6.0 (R Core Team, 2019) [25].

Two sensitivity analyses were conducted. The first evaluated the effect of implementation of PROMs in the subset of patients who were seen in both cohorts and completed CG-CAHPS in both time periods. Outcomes were compared across time using McNemar’s test. The second sensitivity analysis explored whether experience with PROMs impacted visit satisfaction in the post-implementation cohort only, comparing patients who completed PROMs with those who did not. Multivariable mixed effects logistic models were constructed as described above, but the cohort predictor was replaced with an indicator of whether the patient completed PROMs. Interaction effects were evaluated in a similar manner to that described above.

## Results

**Fig. 1:**
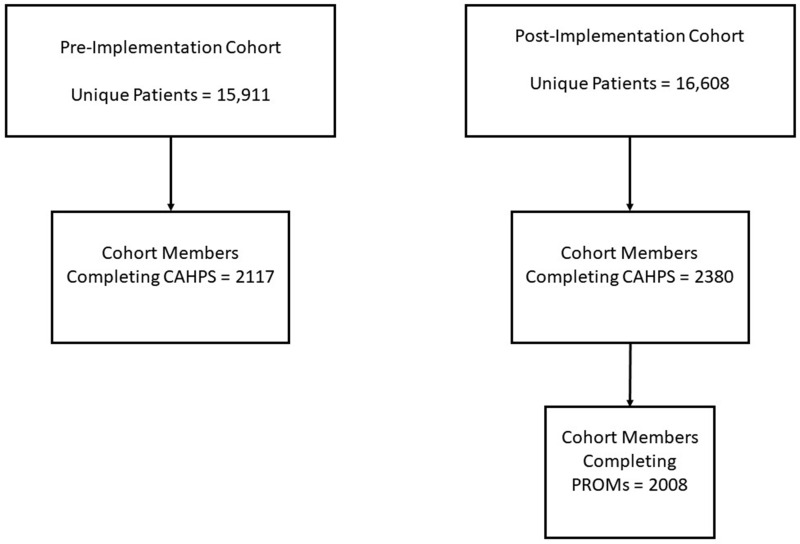
Flow chart of study cohorts CAHPS: Consumer Assessment of Healthcare Providers and Systems; PROMS: patient-reported outcome measures

Between January and June, 15 911 and 16 608 patients were seen in the Rheumatology Department, in the pre-implementation (2016) and post-implementation (2017) periods, respectively (Fig. 1). Of these patients, 2117 completed CG-CAHPS in the pre-implementation cohort and 2380 in the post-implementation cohort. Of those in the post-implementation cohort, 2008 (84%) had PROMs data. There were 486 patients with data in both cohort periods. Table 1 presents demographics and clinical characteristics for both groups. Although statistically different, age and BMI were similar between the pre-implementation and post-implementation cohorts [62.76 (s.d.=13.29) *vs* 61.62 (13.41) years; and median BMI 27.97 *vs* 28.70 kg/m^2^, respectively). The pre-implementation patients were more likely to have a history of cancer (49.6 *vs* 45.7%) compared with post-implementation patients. The post-implementation cohort had a slightly greater percentage of new patients (19.3 *vs* 22.6%) compared with the pre-implementation cohort.

**Table 1:** Characteristics of study cohorts pre- and post- implementation of patient-reported outcome measures

Characteristics	Pre-implementation cohort	Post-implementation cohort	*P*-value
	*n* = 2117	*n* = 2380	
Female sex, *n* (%)	1562 (73.8)	1778 (74.7)	0.502
New patients (*vs* established patients), *n* (%)	408 (19.3)	537 (22.6)	0.008
Non-white, *n* (%)	210 (10.0)	277 (11.7)	0.074
Age, mean (s.d.), years	62.76 (13.29)	61.62 (13.41)	0.004
Age, median [Q1, Q3], years	64.11 [55.06, 71.71]	63.22 [53.86, 70.87]	0.004
Married, *n* (%)	1469 (70.5)	1610 (68.8)	0.245
College degree or higher^a^, *n* (%)	900 (43.0)	1021 (43.3)	0.863
Household income (per $10 000), median [Q1, Q3]	5.76 (1.80)	5.80 (1.89)	0.483
BMI, median [Q1, Q3], kg/m^2^	27.97 [24.37, 33.16]	28.70 [24.70, 33.75]	0.013
Charlson score, mean (s.d.)	2.11 (2.11)	2.04 (2.16)	0.276
Charlson score, median [Q1, Q3]	1.00 [1.00, 3.00]	1.00 [1.00, 3.00]	0.035
Diabetes, *n* (%)	282 (13.3)	291 (12.2)	0.292
Cancer, *n* (%)	1049 (49.6)	1088 (45.7)	0.011
Depression, *n* (%)	527 (24.9)	597 (25.1)	0.910
Hypertension, *n* (%)	983 (46.4)	1059 (44.5)	0.203
Low QOL^b^, *n* (%)	1437 (68.3)	1659 (70.0)	0.230
PROMIS GH physical, mean (s.d.)		43.89 (8.91)	
PROMIS GH mental, mean (s.d.)		48.56 (9.20)	
PROMIS fatigue, mean (s.d.)		54.39 (10.18)	
PROMIS pain interference, mean (s.d.)		56.42 (8.66)	
PROMIS physical function, mean (s.d.)		42.76 (8.46)	
RAPID3, mean (s.d).		9.74 (6.24)	

a Based on CG-CAHPS.

b Single item rating of overall health from CG-CAHPS, categorized as low if response = good, fair or poor. CG-CAHPS: Clinician and Group Practice Consumer Assessment of Healthcare Providers and Systems; GH: global health; PROMIS: patient-reported outcomes measurement information system; Q: quartile; QOL: quality of life; RAPID3: routine assessment of patient index data 3.

The outcomes and unadjusted ORs by cohort are presented in Table 2. Top box responses ranged from 88.2 to 95.1% in the pre-implantation cohort and from 88.6 to 96.0% in the post-implementation cohort. The only statistically significant difference between cohorts was on the communication domain question of whether the patient’s ‘concerns [were] answered with easy to understand information’ [91.51% in pre- *vs* 93.75% in post-implementation cohort; OR = 1.68 (95% CI: 1.14, 2.48) for the post-implementation cohort]. There were no significant or meaningful differences between the pre- and post-implementation cohorts on the other communication items. Likewise, none of the three primary outcome measures (provider rating, provider recommendation and composite satisfaction with care) was significantly different between cohorts. Composite satisfaction was 85.77 and 85.62% for the pre- and post-implementation cohorts, respectively.

**Table 2:** Frequency of top box responses by item and global measures with unadjusted odds ratios

Outcomes^a^	Pre-implementation cohort	Post-implementation cohort	Unadjusted odds ratio (95% CI)	*P*-value
	*n* (%)	*n* (%)		
Communication domain questions				
Explained things in a way that was easy to understand	1853 (94.01)	2097 (94.37)	1.08 (0.75, 1.56)	0.681
Listened carefully	1860 (94.37)	2100 (94.59)	1.11 (0.72, 1.71)	0.646
Concerns answered with easy to understand information	1736 (91.51)	2009 (93.75)	1.68 (1.14, 2.48)	0.009
Knew important information about medical history	1778 (90.48)	1997 (90.12)	1.30 (0.6, 2.81)	0.506
Showed respect for what you had to say	1878 (95.09)	2134 (95.95)	1.43 (0.89, 2.29)	0.137
Spent enough time	1864 (94.57)	2099 (94.51)	0.99 (0.72, 1.36)	0.950
Global measures				
Provider rating 9 or 10	1729 (88.21)	1963 (88.62)	1.11 (0.82, 1.5)	0.494
Recommend provider's office to family/friends	1813 (92.36)	2081 (93.53)	1.44 (0.94, 2.19)	0.094
Overall satisfaction with care	1675 (85.77)	1881 (85.62)	1.02 (0.76, 1.37)	0.912

a Odds ratios are presented for the post-implementation cohort (with the pre-implementation cohort as the referent). Satisfaction with care is a composite measure defined as a top box response to Clinician and Group Practice Consumer Assessment of Healthcare Providers and Systems (CG-CAHPS) communication domain questions of provider listened, showed respect, spent enough time, and a provider rating of 9 or 10. Sample size varied between items owing to missing values.

Multivariable mixed effects logistic regression models were constructed for the three primary outcomes of provider rating, provider recommendation and composite satisfaction (Table 3). For all three outcomes, there were no main effects for post- *vs* pre-implementation cohort. However, being a new patient was related to lower provider rating [OR = 0.56 (95% CI: 0.43, 0.73)], lower provider recommendations [OR = 0.70 (95% CI: 0.50, 0.98)] and lower overall satisfaction with care [OR = 0.60 (95% CI: 0.47, 0.77)]. Older patients were more likely to give a top box provider rating [OR = 1.29 (95% CI: 1.15, 1.44)], top box provider recommendations [OR = 1.56 (95% CI: 1.34, 1.81)] and higher satisfaction with care [OR = 1.19 (95% CI: 1.07, 1.32)]. Female patients were less likely to give a top box provider rating [OR = 0.70 (95% CI: 0.53, 0.92)], to recommend the provider [OR = 0.43 (95% CI: 0.29, 0.64] and to report satisfaction with care [OR = 0.68 (95% CI: 0.53, 0.87)]. Married patients were more likely to report higher overall satisfaction with care [OR = 1.26 (95% CI: 1.01, 1.56)]. Finally, patients with low QOL were less likely to give a top box provider rating [OR = 0.49 (95% CI: 0.37, 0.65)], less likely to give a top box provider recommendation [OR = 0.43 (95% CI: 0.30, 0.61)] and less likely to report overall satisfaction with care [OR = 0.48 (95% CI: 0.37, 0.61)]. There were no interactions between cohort and any of the covariates on the three outcome measures.

**Table 3:** Multivariable models for predicting provider rating, provider recommendation and satisfaction with care

	Model 1: provider rating		Model 2: provider recommendation		Model 3: overall satisfaction with care	
	*n* = 4029		*n* = 4040		*n* = 4007	
Characteristics	Odds ratio (95% CI)	*P*-value	Odds ratio (95% CI)	*P*-value	Odds ratio (95% CI)	*P*-value
Cohort	1.05 (0.84, 1.31)	0.668	1.20 (0.91, 1.60)	0.204	0.97 (0.79, 1.19)	0.765
New patient (*vs* established patient)	0.56 (0.43, 0.73)	<0.001	0.70 (0.50, 0.98)	0.039	0.60 (0.47, 0.77)	<0.001
Age (per year)	1.29 (1.15, 1.44)	<0.001	1.56 (1.34, 1.81)	<0.001	1.19 (1.07, 1.32)	<0.001
Female (*vs* male)	0.70 (0.53, 0.92)	0.009	0.43 (0.29, 0.64)	<0.001	0.68 (0.53, 0.87)	<0.001
Married (*vs* non-married)	1.25 (0.98, 1.58)	0.068	0.89 (0.66, 1.21)	0.458	1.26 (1.01, 1.56)	0.039
Non-white (*vs* white)	0.89 (0.64, 1.24)	0.495	1.13 (0.72, 1.75)	0.600	1.00 (0.73, 1.38)	0.997
Income (per $10 000)	1.00 (0.94, 1.06)	0.941	0.95 (0.88, 1.03)	0.191	1.00 (0.95, 1.06)	0.928
College degree or higher (*vs* <college)^a^	0.99 (0.78, 1.24)	0.897	1.08 (0.81, 1.44)	0.606	0.99 (0.8, 1.22)	0.939
Charlson score	1.01 (0.95, 1.06)	0.832	1.01 (0.94, 1.09)	0.696	1.01 (0.96, 1.07)	0.618
Low QOL^b^	0.49 (0.37, 0.65)	<0.001	0.43 (0.30, 0.61)	<0.001	0.48 (0.37, 0.61)	<0.001

Cohort odds ratios are presented for the post-implementation cohort (with the pre-implementation cohort as the referent). Satisfaction with care is a composite measure defined as a top box response to CG-CAHPS communication domain questions of provider listened, showed respect, spent enough time, and a provider rating of 9 or 10.

a Based on CG-CAHPS.

b Single item rating of overall health from CG-CAHPS, categorized as low if response = good, fair or poor. CG-CAHPS: Clinician and Group Practice Consumer Assessment of Healthcare Providers and Systems; QOL: quality of life.

A sensitivity analysis was conducted in the 486 patients who had outcome data in both cohorts. Patients had increased top box responses for recommending the provider from pre- (95%) to post-implementation (97%) (*P* = 0.09). Responses were similar across the other outcomes (data not shown).

**Fig. 2:**
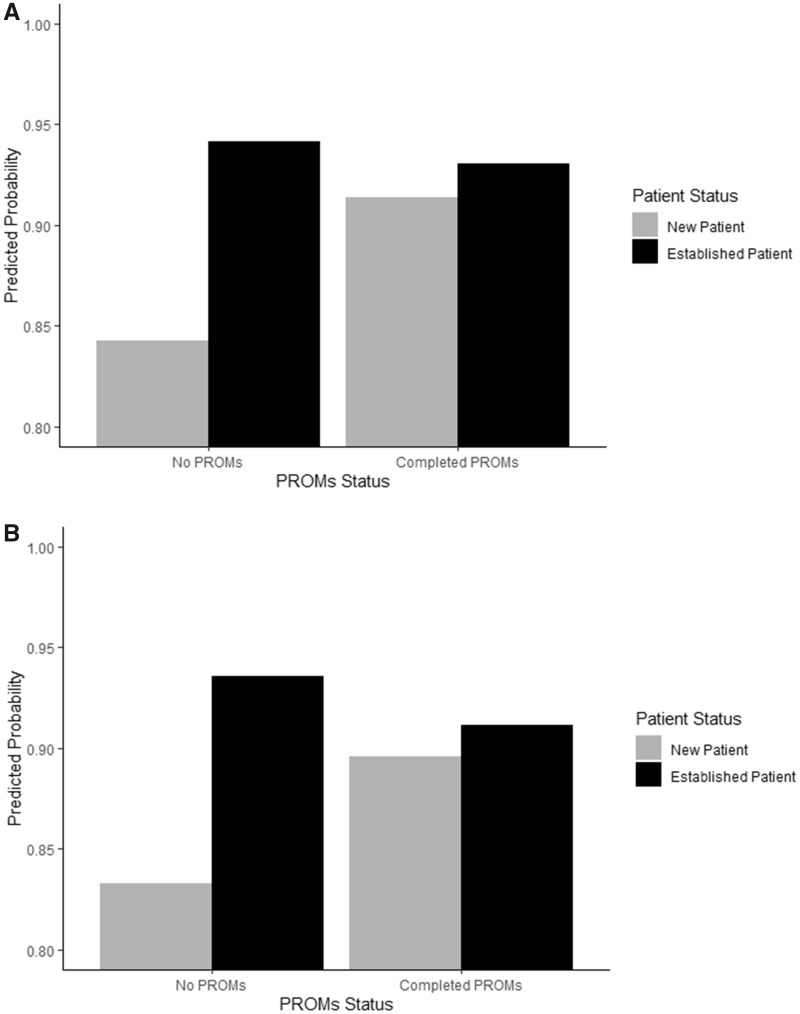
Predicted probability of satisfaction measures for patient-reported outcome measures completion status by new *vs* established patients Predicted probabilities from multivariable mixed effects models (Table 4) for top box provider rating (**A**) and overall satisfaction with care (**B**) for established and new patients completing *vs* not completing PROMs. PROMS: patient-reported outcome measures.

A second sensitivity analysis assessed differences in patients who did *vs* did not complete PROMs in the post-implementation cohort (Table 4). Of the 2380 patients in the post- implementation cohort, 2008 (84.4%) completed PROMs, whereas 372 did not complete PROMs. In multivariable mixed effects logistic regression models, there was no effect for completing PROMs on the global outcomes. Predictors for the three primary global outcomes in the post-implementation cohort were similar to those from both cohorts, with older age positively predicting greater satisfaction for all outcomes and low QOL negatively predicting satisfaction. Additionally, new patients negatively predicted top box scores in provider rating and overall satisfaction with care compared with established patients. However, there were significant interaction effects between PROMs by new patient status for both provider rating and overall satisfaction (Fig. 2). For both interactions, completing PROMs was related to a significant increase in top box scores in new patients, whereas established patients had high scores independent of completing PROMs.

**Table 4:** Multivariable models for predicting provider ratings, provider recommendation and overall satisfaction in the post-implementation cohort

	Model 1: provider rating		Model 2: provider recommendation		Model 3: overall satisfaction with care	
	*n* = 2145		*n* = 2153		*n* = 2130	
Characteristics	Odds ratio (95% CI)	*P*-value	Odds ratio (95% CI)	*P*-value	Odds ratio (95% CI)	*P*-value
Completed PROMs	0.83 (0.52, 1.34)	0.453	1.18 (0.70, 1.98)	0.534	0.71 (0.46, 1.10)	0.126
New patient (*vs* established patient)	0.33 (0.13, 0.81)	0.015	0.98 (0.64, 1.52)	0.942	0.34 (0.14, 0.78)	0.012
Age (per year)	1.35 (1.17, 1.56)	<0.001	1.61 (1.34, 1.94)	<0.001	1.19 (1.05, 1.36)	0.007
Female (*vs* male)	0.85 (0.60, 1.19)	0.339	0.63 (0.39, 1.00)	0.052	0.80 (0.59, 1.09)	0.163
Married (*vs* non-married)	0.98 (0.72, 1.33)	0.906	0.79 (0.53, 1.17)	0.238	1.07 (0.81, 1.41)	0.626
Non-white (*vs* white)	0.90 (0.59, 1.37)	0.625	1.14 (0.65, 2.01)	0.649	0.99 (0.66, 1.47)	0.948
Income (per $10 000)	0.95 (0.88, 1.03)	0.225	0.93 (0.84, 1.03)	0.151	0.97 (0.90, 1.04)	0.385
College degree or higher (*vs* <college)^a^	0.87 (0.65, 1.17)	0.362	0.95 (0.65, 1.38)	0.787	0.94 (0.72, 1.22)	0.637
Charlson score	1.03 (0.96, 1.10)	0.473	1.08 (0.97, 1.19)	0.161	1.04 (0.97, 1.11)	0.234
Low QOL^b^	0.52 (0.37, 0.74)	<0.001	0.49 (0.31, 0.77)	0.002	0.53 (0.39, 0.72)	<0.001
Interaction: completed PROMs×new patient	2.4 (0.93, 6.19)	0.070	–	–	2.46 (1.01, 6.00)	0.047

a Based on CG-CAHPS.

b Single item rating of overall health from CG-CAHPS, categorized as low if response = good, fair or poor. CG-CAHPS: Clinician and Group Practice Consumer Assessment of Healthcare Providers and Systems; PROMS: patient-reported outcome measures, QOL: quality of life.

## Discussion

Our study found limited evidence of the effect of PROMs on visit satisfaction, as measured by three global outcomes: overall provider rating on a 10-point scale, the patient’s report of willingness to recommend the caregiver to family, and a composite measure of overall satisfaction with care, indicated by a high provider rating and a high response to the items provider listened, showed respect and spent enough time. Responses on these three global measures were extremely high in both cohorts (88–94%). Responses to communication items on the CG-CAHPS were also high in our study (90–96%). However, other studies of patient experience have found that CG-CAHPS ratings of communication questions tend to be high. A study of 21 318 patients across 450 practice sites demonstrated communication top box scores ranging from 90 to 95%, with provider ratings from 82 to 90% [23]. In rheumatology patients, responses have been shown to be similarly high, with a study of >2800 rheumatology patients having communication and provider ratings ranging from 92 to 95% [26]. The implementation of PROMs into an office visit has the potential to be burdensome for patients and to cause disruption to the clinical workflow for providers and office staff. Our study found that visit satisfaction remained high among established patients in an ambulatory setting and that new patients demonstrated higher levels of satisfaction after implementation of PROMs.

Although implementation of PROMs did not predict higher responses in any of the three primary outcome measures in our study, other predictors were shown to have an effect on visit satisfaction. Sensitivity analyses showed that for patients new to the Rheumatology Center, completing PROMs was a significant predictor of higher top box scores in provider rating and overall satisfaction with care. However, this comparison is somewhat limited, owing to a very low percentage of patients who did not complete PROMs. Overall, being a new patient was related to less satisfaction, as was having a low quality of life and being female. However, older age was related to better scores on all three outcomes. Established patients, higher quality of life and older age have been demonstrated in the literature to predict increased satisfaction with medical visits [27–29]. The relationship between sex and visit satisfaction has been largely contradictory [29], with female sex and increased visit satisfaction being shown in some studies [30], but not in others [27, 28, 31]. In rheumatology patients specifically, older age and follow-up visits have also been associated with higher visit satisfaction [32]. A cohort study of 573 rheumatology patients determined predictors of high and low satisfaction based on quartiles and, in contrast to our study, found that female sex was associated with higher visit satisfaction. Another study in patients with RA found that women were more likely to rate certain visit aspects significantly better than men but that sex was largely unrelated to patients’ view on care [33].

As health care in the USA transitions from a fee-for-service to an outcomes-based environment, enhancing the patient experience has become a priority for both policy-makers and clinical leaders. When incorporated into the clinic visit, PROMs could theoretically direct patient-centred care and increase patient satisfaction with their visit and care. PROMs allow clinicians to capture patient views, feelings and subjective experiences. When clinicians are better able to understand a patient’s health from their perspective, it can enhance provider–patient communication, enable shared decision-making and impact how the patient thinks about his condition [34]. Our study suggests that the act of completing PROMs alone might not impact overall communication with the provider or satisfaction with care. The review process is crucial to the effectiveness of PROMs. A mixed methods study found that both patients and providers felt that reviewing PROMs results contributed to increased insight into the patient’s condition and led to shared decision-making [12]. The results of our study might provide an innovative approach to increase patient satisfaction and positively impact patient-centred care if the provider reviews the PROMs with the patient.

For rheumatology patients, including PROMs as part of the office visit might provide important information beyond their physician-led care. As evidence of this, a meta-analyses of 109 clinical trials found that PROMs were useful in a treat-to-target strategy, when compared with usual care [35]. Also, PROMs might better discriminate active from placebo treatment than physician-reported measures in randomized controlled trials. Strand *et al.* [36] found that in two randomized controlled trials with >800 patients with active RA, PROMs of disease activity, pain and physical function showed no improvement for patients in the placebo group. In contrast, physician-reported measures showed improvement for patients with RA.

Our study has a number of strengths. We included a large study of representative patients seen in rheumatology clinics within a large, integrated health system. Given that PROMs were rolled out within a 2-month window to all rheumatology centres, we were uniquely positioned to answer our research question of whether PROMs impacted overall visit satisfaction.

Our study also has a number of limitations. First, the pre–post study design is correlational, and therefore we are unable to assess causality. The act of completing PROMs alone did not show any effect on CG-CAHPS scores, but it is unknown whether providers reviewed PROMs and communicated them to the patient. Future longitudinal studies should assess how to present PROMs to the patient and provider and how to facilitate communication about PROMs in order to optimize engagement. Second, there was high satisfaction across CG-CAHPS items and summary scores. The dearth of unsatisfied patients limits the ability to detect change over time. Third, not all patients in the post-implementation cohort completed PROMs; however, the majority (84%) completed at least some PROMs. We do not know whether the patients who did not complete PROMs were offered PROMs, reviewed them without completing and/or declined participation. Lastly, our study is limited to the proportion of patients who completed CG-CAHPS. The surveys are sent to a random selection of patients, and we controlled for demographics in multivariable models in an attempt to account for possible selection bias. Although our response rate is low (14%), this is a universal issue of concern with CAHPS surveys, as noted in a 2017 review from the Agency for Healthcare Research and Quality (AHRQ) [37]. Despite the potential for response bias, Centers for Medicare and Medicaid Services and private insurers still use CAHPS as the primary measure of patient experience in the USA and have found the results to be a valid source of data from which to base many policy and reimbursement practices.

In conclusion, we found high levels of satisfaction with care in patients seen in rheumatology clinics. Implementation of systematic collection of PROMs within the rheumatology clinics had little demonstrable effect on patient satisfaction with ambulatory care. Further evaluation of the impact of PROMs collection within clinical care should incorporate review of the results with patients into the study design.

*Funding:* No specific funding was received from any funding bodies in the public, commercial or not-for-profit sectors to carry out the work described in this manuscript.

*Disclosure statement:* C.D. receives fees of $5000 or more per year as a paid consultant, speaker or member of an advisory committee for Amgen, Inc. and Alexion Pharmaceuticals, Inc. The other authors have declared no conflicts of interest.

## Data availability statement

The data underlying this article cannot be shared publicly due to the privacy of individuals that participated in the study. The data will be shared on reasonable request to the corresponding author.
